# Integrated BSA-seq and RNA-seq analyses reveal *CmRABE1c* as a negative regulator of drought tolerance and enable the development of a promoter SNP marker in Chinese chestnut

**DOI:** 10.3389/fpls.2026.1884976

**Published:** 2026-07-27

**Authors:** Yan Liu, Yan Guo, Xuan Wang, Haie Zhang, Liyang Yu, Dongsheng Wang, Jing Liu

**Affiliations:** 1 Engineering Research Center of Chestnut Industry Technology, Ministry of Education, Hebei Normal University of Science and Technology, Qinhuangdao, China; 2 Changli Institute of Pomology, Hebei Academy of Agricultural and Forestry Sciences, Changli, China

**Keywords:** BSA-seq, Chinese chestnut, *CmRABE1c*, drought tolerance, functional marker, RNA-Seq

## Abstract

Drought stress severely limits both yield and nut quality of Chinese chestnut (*Castanea mollissima* Blume), an economically and ecologically important woody grain species native to China. However, the genetic basis of drought tolerance in Chinese chestnut remains a research gap. Our study fills this gap by identifying novel drought-responsive genes, which represents the first comprehensive molecular characterization of drought adaptation in this species. To dissect the genetic basis of drought tolerance and develop functional markers, we investigated 316 F1 seedlings derived from the cross between the drought-tolerant ‘Yanlong’ cultivar and the drought-sensitive ‘Shuofeng’. Bulked segregant analysis coupled with next-generation sequencing (BSA-seq) identified 80 drought-associated quantitative trait loci (QTLs) across 12 chromosomes. Integration of BSA−seq, RNA-seq, and parental resequencing resulted in the identification of a core candidate gene, *CmRABE1c*, encoding a ras-related protein. *Cm*RABE1c overexpression in tobacco and poplar confirmed its negative regulatory role in drought tolerance. Notably, a GG-to-AA substitution at -312/-313 bp in the *Cm*RABE1c promoter disrupted the GT1-motif and was significantly associated with drought tolerance across 60 chestnut accessions. Furthermore, transgenic tobacco and poplar assays proved this SNP may be diagnostic by showing that the AA allele confers superior drought tolerance compared to the GG allele. This diagnostic marker likely can be directly applied in marker-assisted selection (MAS) to screen and develop drought-tolerant chestnut cultivars, thereby accelerating breeding efficiency. In summary, this study provides valuable genetic resources and a robust functional SNP marker for drought resistance breeding in Chinese chestnut.

## 1 Introduction

Chinese chestnut (*Castanea mollissima Blume*) is an important woody nut tree species native to China that holds ecological and economic value. In China, *C. mollissima* exhibits the highest nut yield and quality within the Fagaceae family, and plays a significant role in rural poverty alleviation (Ji et al., 2018). However, chestnut orchards are predominantly located in mountainous regions where irrigation infrastructure is limited. Under the escalating impacts of global climate change, the increasing frequency of extreme high temperatures and seasonal drought has rendered drought stress a major abiotic constraint limiting chestnut yield and quality (Lesk et al., 2016). Developing and deploying drought-tolerant cultivars represents the most cost-effective strategy to mitigate these impacts. Yet the genetic architecture and molecular regulation of drought tolerance in chestnut have not been fully elucidated, and molecular markers linked to drought resistance are still lacking. Consequently, clarify the genetic characteristics of drought resistance and developing reliable molecular markers are essential for breeding drought-resilient varieties and ensuring the sustainable development of the chestnut industry.

Drought stress is a complex quantitative trait governed by a combination of major−effect and minor−effect loci. Numerous drought−related QTLs have been identified in cereal crops and woody plants by conventional linkage mapping or GWAS method (de Miguel et al., 2014; Depardieu et al., 2021; Alahakoon and Fennell, 2023; Yue et al., 2026). However, these strategies entail higher costs due to the requirement for larger population sizes and denser marker coverage. Although bulked segregant analysis (BSA-seq) provides a low-cost and rapid alternative for QTL dissection in trees, its application in *Castanea mollissima* remains underexplored (Shen et al., 2019; Liu et al., 2020). Furthermore, while transcriptomic studies have long dominated the identification of drought-responsive genes in chestnut-yielding numerous candidates-the field still lacks effective screening strategies, accurate estimation of effects, and robust *in vivo* functional validation of core candidate genes (Fernandes et al., 2022).

Diagnostic or functional markers developed from causal variants within gene-coding or promoter regions enable precise marker-assisted selection (Salgotra and Stewart, 2020). SNP-based functional markers have advanced drought−tolerance breeding in several woody species. In tea, a KASP-SNP marker targeting *Cs*AGD6 (chr10:206216541 C/T) was developed and validated in 104 accessions, enabling efficient early selection of drought-tolerant genotypes (Yue et al., 2026). In wheat, a novel KASP marker (DE106) derived from the SNP Excalibur_c100336_106 within the major QTL qDSI.4B.1 explained up to 14% of phenotypic variation in drought tolerance (Liu et al., 2023). In *Pinus pinaster*, SNPs within a MYB transcription factor located within drought−associated QTL regions were significantly associated with photosynthetic efficiency, serving as functional markers for MAS (de la Mata et al., 2026). In chestnut, drought-related SSR and EST-SSR markers have been applied mainly to genetic diversity studies, whereas functional SNP markers targeting drought-tolerance genes remain scarce (Alcaide et al., 2019; Perez-Rial et al., 2025). Such functional SNP markers, derived from causal coding or promoter regions, provide robust tools for MAS, accelerating the breeding of drought-resistant chestnut varieties.

To address these limitations, we employed BSA-seq combined with next-generation resequencing to map drought-responsive QTLs. Subsequently, we integrated BSA-seq, RNA-seq, and parental resequencing data to predict and prioritize candidate genes, followed by molecular assays to develop and validate functional markers. Specifically, the objectives of this study were to: (1) map genome-wide drought-responsive QTLs using an F1 segregating population derived from *Castanea mollissima* cultivars; (2) identify key candidate genes through multi-omics integration; (3) validate gene function via heterologous transgenic assays; and (4) develop functional SNP markers for drought-resistant marker-assisted selection. This work is expected to lay a solid genetic foundation and assist breeding for drought stress tolerance in perennial crops such as chestnut.

## 2 Materials and methods

### 2.1 Plant materials and drought treatments

The experiment was conducted using 316 F_1_ hybrid chestnut seedlings derived from a cross between the drought−tolerant cultivar ‘Yanlong’(‘YL’) and the drought−sensitive cultivar ‘Shuofeng’(‘SF’). The parental genotypes and all hybrid progeny were maintained by Hebei Normal University of Science and Technology. Controlled pollination of ‘YL’ × ‘SF’ was performed in 2021, and the resulting hybrid seeds were germinated and sown in the spring of 2022, yielding a total of 316 hybrid seedlings. All seedlings were grown in pots (38cm diameter)filled with 3:1:1 peat-perlite-vermiculite substrate in a greenhouse with drip irrigation using half−strength Hoagland nutrient solution. Environmental parameters were continuously monitored throughout the experiment. The plastic greenhouse was well ventilated and fully illuminated without shading stress, with air temperature controlled at 22-32°C and relative humidity maintained at 55%–75%. Diurnal fluctuations of temperature and humidity remained stable, and no extreme environmental stress occurred. All potted seedlings were arranged uniformly and rotated regularly to mitigate the impacts of microenvironmental heterogeneity on plant growth and drought-associated phenotypes. The saturated water content of the cultivation substrate was 48.6%. Under normal water supply, soil moisture was kept at 75%-80% of the maximum field capacity.

In the springs of 2023 and 2024, the hybrid seedlings were fully irrigated to field capacity, and subsequently subjected to a 49−day drought treatment. Throughout the treatment period, soil moisture content was dynamically monitored to ensure consistent drought stress levels, the measurement was performed according to previously protocol (Berauer et al., 2024). Hybrid seedling responses were recorded at 7−day intervals, and the drought injury was quantitatively assessed on a 0–5 scale based on visual morphological symptoms, including the severity of leaf wilting, curling, discoloration, and defoliation, according to established criteria (Zeng et al., 2024). The drought injury index (H) was calculated as a weighted average of injury grades at 35, 42, and 49 days using the formula: H = 0.25×H_35_ + 0.60×H_42_ + 0.15×H_49_(H_x_ = injury grade at day X). The weighting coefficients were determined based on phenotypic distributions and empirical optimisation. At 35 d, distribution was severely left-skewed (>85% individuals in grades 0-1), offering limited genetic resolution; at 49 d, it was right-skewed (>68% in grades 4-5), dominated by physiological noise. In contrast, 42 d approached normality (~80% intermediate grades), maximising genetic variance and enabling accurate resistant/susceptible discrimination. We tested multiple weight combinations; the selected (0.25, 0.60, 0.15) gave the highest cross-year reproducibility of extreme F_1_ individuals, confirming robustness.

### 2.2 Bulked segregant analysis sequencing

In 2024, hybrid offspring exhibiting stable drought injury index over two consecutive years (P > 0.05) were identified. Based on this index, 50 extremely drought-tolerant and 50 extremely drought-sensitive plants were selected to construct the tolerant (T) and sensitive (S) bulks, respectively. To characterize and validate the physiological differences between these two contrasting groups, we assessed various phenotypic parameters of the selected individuals within these bulks on the 49th day of the drought treatment. These assessments encompassed plant height and stem girth (growth traits); malondialdehyde (MDA) and hydrogen peroxide (H_2_O_2_) contents, as well as peroxidase (POD) activity (biochemical traits); and stomatal conductance, transpiration rate, and net photosynthetic rate (photosynthetic traits). All the photosynthetic measurements were performed according to previously published protocols (Khalid et al., 2021; Fu et al., 2025).

Genomic DNA was then extracted, pooled in equimolar amounts, and sequenced on an Illumina HiSeq X Ten platform (Illumina, San Diego, CA, USA) at approximately 30× depth. Clean reads were then aligned to the ‘Yanbao’ reference genome using the Burrows-Wheeler Alignment software (Li and Durbin, 2010; Daccord et al., 2017). Allelic differentiation between bulks was assessed using the G’-value method in BSATOS v2.0 (Magwene et al., 2011; Shen et al., 2019). Quantitative trait loci (QTL) allelic variations were classified by integrating parental resequencing data into three categories (M: maternal heterozygous, paternal homozygous; P: paternal heterozygous, maternal homozygous; H: both parents heterozygous). Significance thresholds for G’ statistic were determined by BSATOS software. The genome-wide significance thresholds(q<0.01) were established separately for QTLs derived from different parental origins: 3.2 for maternal-origin QTLs (M prefix) and 4.83 for paternal-origin QTLs (P prefix).

### 2.3 RNA-seq analysis

Transcriptome datasets used in this study were derived from our previous research, comprising three independent biological replicates per condition (Wang et al., 2024).The exon model fragments per kilobase per million mapped fragments (FPKM) were calculated using TopHat v2.1.1 and HTSeq v0.11.3 with Perl scripts. To ensure reliability, genes with a mean FPKM > 10 were retained. Differentially expressed genes (DEGs) were identified using DESeq2, with thresholds of |log_2_(fold change)| ≥ 0.5 and P < 0.05. Weighted gene co-expression network analysis (WGCNA) of the DEGs was performed using the WGCNA R package (Da et al., 2019). The core parameters were configured as: soft-thresholding power = 18 (R^2^ cutoff = 0.80), unsigned network type, maxBlockSize = 20,000, deepSplit = 2, minModuleSize = 30, and mergeCutHeight = 0.25.

### 2.3 Candidate gene prediction

Functional annotation of candidate genes was performed using UniProt (http://www.uniprot.org) and the eggNOG database. Gene Ontology (GO) and Kyoto Encyclopedia of Genes and Genomes (KEGG) enrichment analyses were visualized using TBtools software and the OmicShare online platform (https://www.omicshare.com/).

### 2.4 Gene cloning and expression analysis

Genomic DNA was extracted from the young leaves of the two parents using a modified CTAB method (Porebski et al., 1997). Total RNA was isolated using the Polysaccharide-Polyphenol Plant Total RNA (Plus) Small Volume Extraction Kit (SinoBio Technology Co., Ltd). Primers were designed using the Primer5 software based on the target sequence. High−fidelity PCR amplification was performed using Platinum Taq DNA Polymerase (Thermo Fisher Scientific) with specific primers to clone promoter regions. The PCR-amplified fragments were sequenced by Sangon Biotech (Shanghai) Co., Ltd. (Shanghai, China; https://www.sangon.com). The obtained sequences were aligned to the ‘Yanbao’ reference genome to identify polymorphic sites. Primers for real-time quantitative PCR (RT-qPCR) were designed accordingly. RT-qPCR was conducted using SYBR Premix Ex Taq (TaKaRa) on an ABI 7500 RT-PCR system (Applied Biosystems). β-ACTIN was used as the internal reference gene. All primers used in this study are listed in **Supplementary Table 15**.

### 2.5 Functional validation of the candidate gene

The coding sequence of the target gene was inserted into an expression vector and transformed into competent *Agrobacterium tumefaciens* GV3101 cells. Subsequently, the recombinant strain was used to transform *Nicotiana benthamiana* and *Populus tomentosa*, respectively, following established protocols (Yan et al., 2020; Liu et al., 2021; Niedbala et al., 2021). After two days of co-cultivation, tobacco leaf explants were transferred to callus induction medium and incubated for approximately 10 days to induce callus formation. Uniform calli with robust growth were selected and subcultured on selection medium for 15–30 days at a constant temperature of 23 ± 2°C. Subsequently, vigorously growing positive calli were transferred to differentiation medium and cultured for an additional 15–30 days. Similarly, transgenic poplar explants were co−cultivated for three days, followed by 25 days on selection medium. Root induction was performed on 1/2 MS medium supplemented with 0.2 mg/L NAA, with a culture duration of 15 days. Regenerated plantlets were then transferred to seedling-strengthening medium for 7–10 days. Well-developed plantlets were carefully rinsed to remove residual medium from the root system and transplanted into a substrate mixture of nutrient soil, perlite, wormcast, and vermiculite at a volume ratio of 5:0.5:1.5:1. After 20 days of conventional management, all plantlets were irrigated to soil saturation and then subjected to drought treatment for 14 days (tobacco) or 6 days (poplar). Each drought treatment was performed with at least three independent replicates of transgenic seedlings, and a minimum of 9 seedlings were used for each replicate.

### 2.6 Marker development of SNP 312/313

To genotype the *CmRABE1c-*SNP 312/313, 60 *Castanea* accessions were analyzed using Sanger sequencing.

### 2.7 Statistical analysis

All statistical analyses were performed using SPSS software. The normality of data distribution and homogeneity of variances were tested prior to Student’s t-test. Bonferroni correction was used for multiple comparisons to adjust the significance threshold. The broad-sense heritability (H²) was calculated using the formula: H² = (MSg − MSe)/MSg, MSg Means square of genotype/group, MSe means square of error.

## 3 Results

### 3.1 Phenotypic segregation in hybrid chestnut seedlings

**Figure 1:**
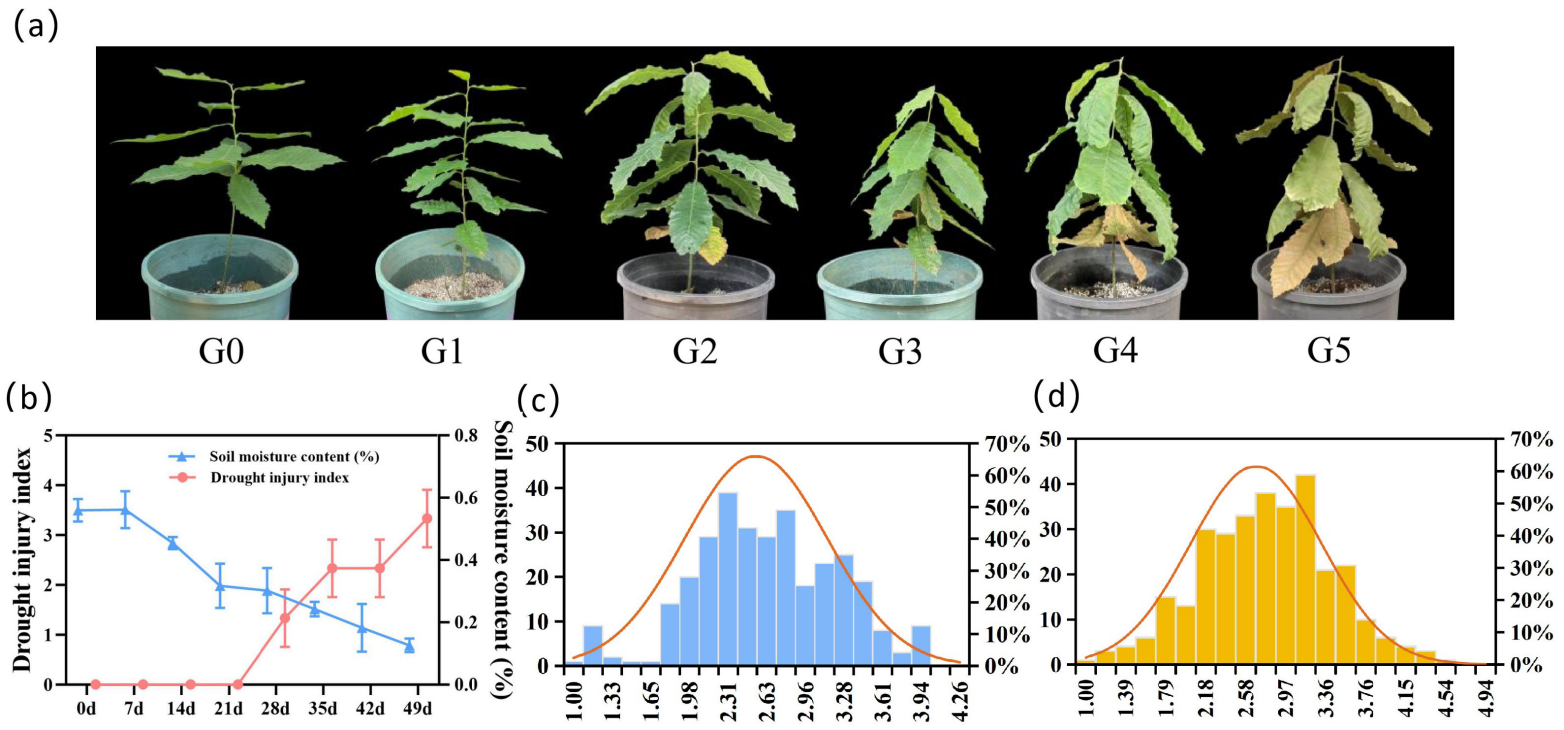
Phenotypic responses of hybrid chestnut seedlings to progressive drought stress, and distribution of drought injury indices. **(a)** Representative phenotypes of hybrid chestnut seedlings exhibiting different drought injury grades (G0–G5) after 49 days of drought stress. G0 = no visible damage; G1–G5 represent increasing severity of leaf wilting, scorching, and necrosis. **(b)** Temporal dynamics of soil moisture content (blue line, left axis) and drought injury index (red line, right axis) over the 49-day drought treatment period. **(c, d)** Frequency distribution of drought injury indices in the hybrid population. Blue and yellow bars represent 2023 and 2024, respectively.

Throughout the 49−day drought treatment, soil moisture in the potting substrate declined progressively, and drought injury intensified with increasing drought stress duration (**Figure 1b**). Visible drought symptoms first appeared on the 15th day of drought stress, characterized by leaf wilting and slight scorching at some leaf tips. By day 35, seedlings within the population displayed grade 4 drought injury symptoms, and clear phenotypic segregation within the population became evident. At the endpoint (day 49), the hybrid seedlings in grade-5 injury class exhibited injury indices ranging from 25.6% to 36.0%(**Figure 1a**).

Across two consecutive years of drought stress treatments, the drought injury index in the hybrid population exhibited a normal distribution, as confirmed by the Shapiro-Wilk test. ANOVA revealed highly significant differences among the 316 individual seedlings (F_315,316_ = 53.70, P < 0.001). Consistent with the strong genetic control, the year-to-year correlation coefficient between 2023 and 2024 was remarkably high (r = 0.989), and the estimated broad-sense heritability was H² = 0.98. Collectively, these results indicate that drought tolerance in chestnut is a typical polygenic quantitative trait under strong genetic control, exhibiting minimal environmental interference and high phenotypic stability across years (**Figures 1c, d**; **Supplementary Table 1**).

### 3.2 Phenotypic evaluation of extreme bulks and QTL identification

**Figure 2:**
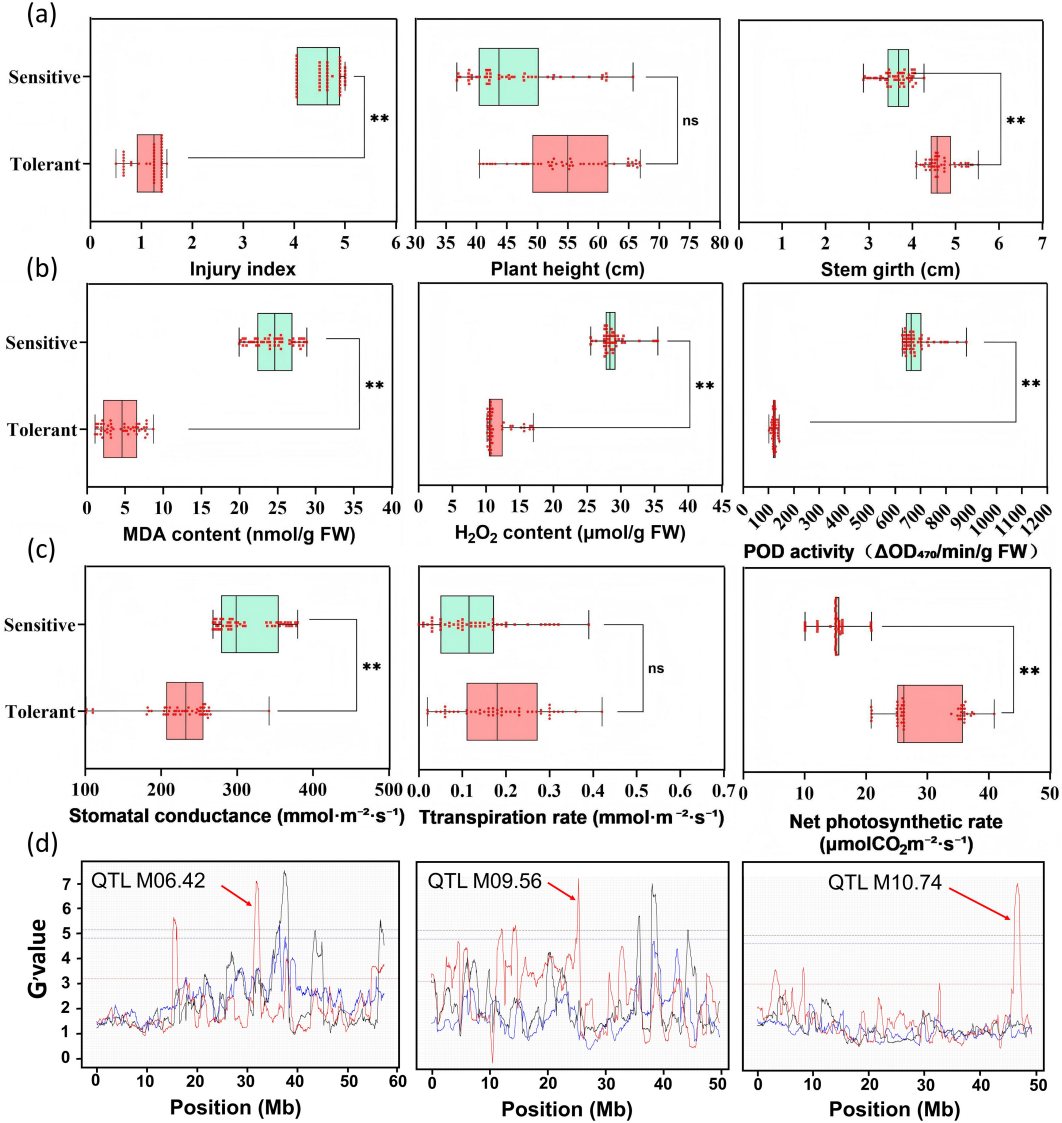
Phenotypic differentiation between drought-tolerant and drought-sensitive bulks and QTL identification via BSA-seq. **(a–c)** Boxplots illustrating phenotypic differences in drought tolerance-related traits between the tolerant (T-bulk, red boxes) and sensitive (S-bulk, green boxes) bulks. **(a)** Agronomic traits: drought injury index, plant height, and stem girth. **(b)** Physiological and biochemical traits: MDA content, H_2_O_2_ content, and POD activity. **(c)** Photosynthetic traits: stomatal conductance, transpiration rate, and net photosynthetic rate. **(d)** G’-value plots of three major QTLs significantly associated with drought tolerance. The symbol ** indicates P < 0.01, ,and ns indicates no significant difference (P > 0.05).

Based on the injury index under drought stress, the 50 most tolerant or sensitive plants were selected to construct tolerant (T) and sensitive (S) bulks. The S−bulk exhibited significantly higher average drought injury indices, malondialdehyde content (MDA), hydrogen peroxide content (H_2_O_2_), peroxidase (POD) activity, and stomatal conductance compared with the T−bulk (p<0.01).In contrast, stem girth, transpiration rate, and net photosynthetic rate were markedly higher in the T−bulk (**Figures 2a–c**). Sequencing of the two bulk DNA samples generated a total of 183.99 Gb of clean reads, with each bulk contributing more than 27.19 Gb. Sequencing depths ranged from 37.50 to 46.93, and alignment rates to the reference genome exceeded 89.93% (**Supplementary Table 2**). Eighty QTLs were mapped across all 12 chromosomes using BSATOS software. QTLs were particularly enriched on chromosomes 1, 11, and 3, which harbored 8, 7, and 6 QTLs, respectively. Among these, 31 major QTLs with thresholds exceeding 30% were broadly distributed across all 12 chromosomes, with a pronounced clustering observed on chromosomes 9 and 5. Three QTLs (M06.42, M09.56, and M10.74) exhibited significantly elevated G’values, indicating strong associations with drought tolerance (**Figure 2d**; **Supplementary Figure 1**; **Supplementary Table 3**).

### 3.3 Differential expression and functional enrichment of drought-responsive genes

**Figure 3:**
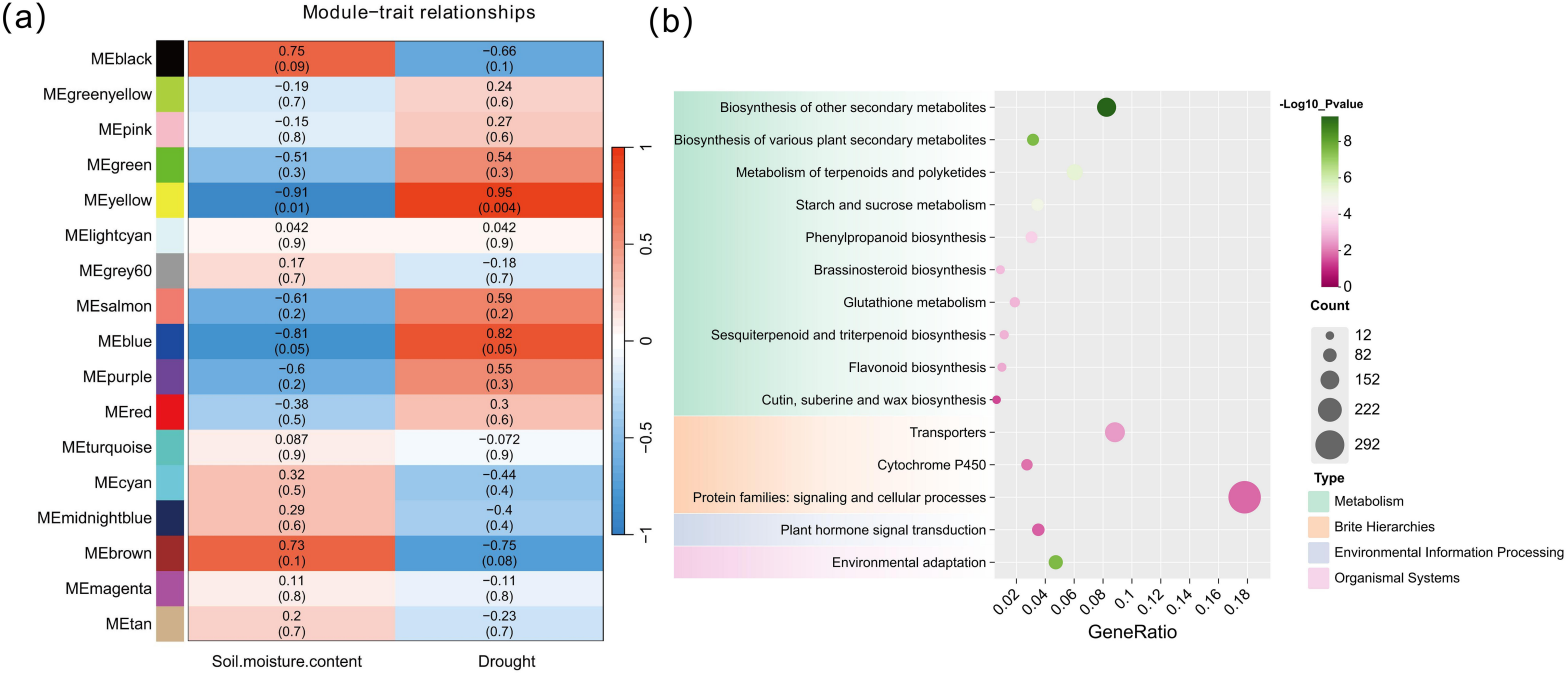
WGCNA module-trait associations and functional enrichment analysis of drought-responsive genes. **(a)** Module-trait correlation matrix generated by WGCNA. The MEyellow module exhibits a strong positive correlation with drought response (r = 0.95, P < 0.01). **(b)** KEGG pathway enrichment analysis of drought-induced DEGs.

**Figure 4:**
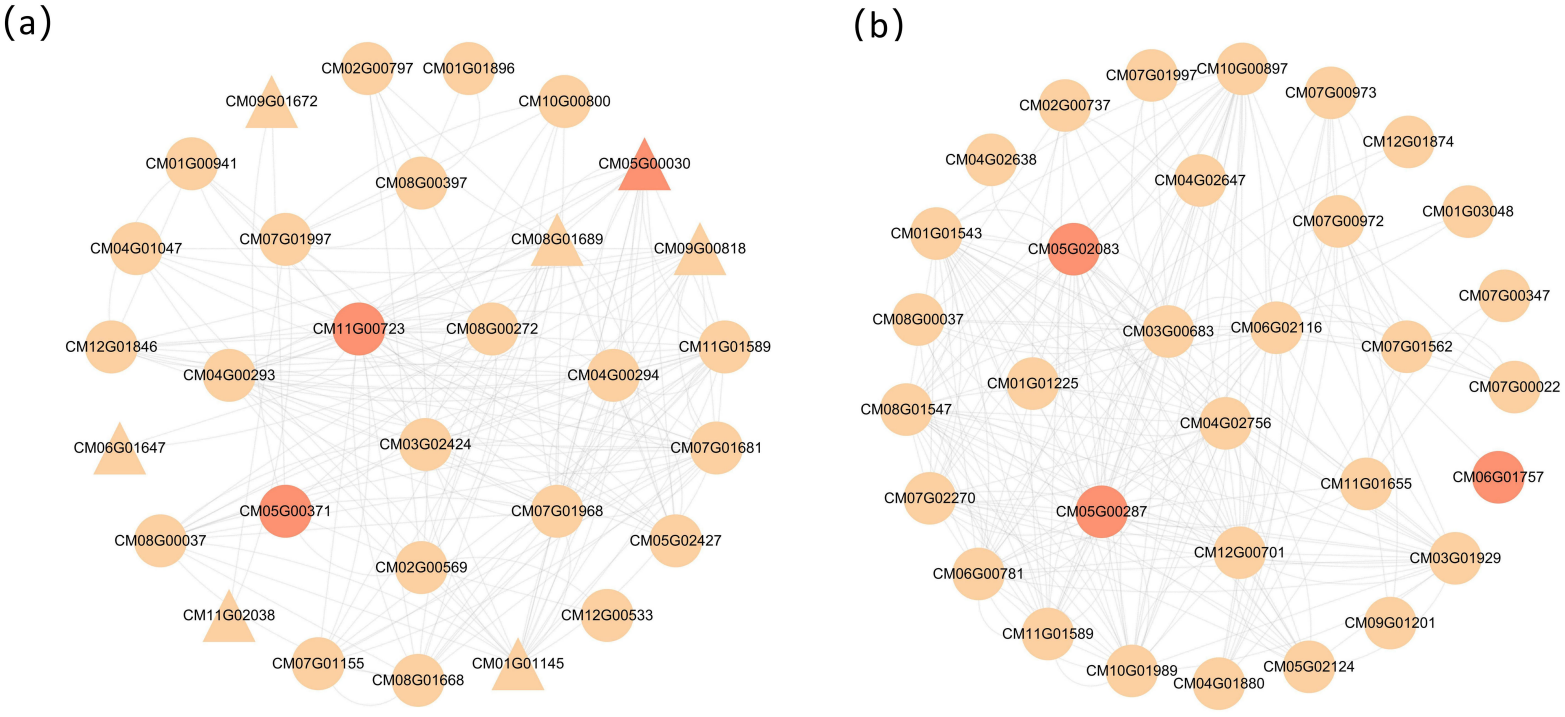
Co-expression networks of drought resistance-related candidate genes in chestnut. **(a, b)** Two representative co-expression networks constructed from drought−responsive DEGs, network of core genes identified through GO enrichment analysis (left, **a**) and network of core genes identified through KEGG enrichment analysis (right, **b**). Orange circles represent genes, and orange triangles represent transcription factors. Darker orange nodes indicate hub genes that overlap with QTL intervals and were prioritized as core candidates.

Using DESeq2, we identified 3,996 significantly differentially expressed genes (DEGs) between drought-stressed and well-watered control plants (thresholds:|log_2_(fold change)| ≥ 0.5 and P < 0.05), including 1,926 up-regulated and 2,070 down-regulated genes (**Supplementary Table 4**). Gene Ontology (GO) enrichment revealed strong representation of pathways associated with osmotic adjustment, ABA signaling, and water deficit response. Cellulose biosynthesis and drought−induced metabolic processes were predominantly overrepresented, with suberin biosynthesis alone comprising 332 genes (**Supplementary Figure 2**; **Supplementary Table 5**). KEGG pathway analysis corroborated these findings, highlighting secondary metabolite biosynthesis as the most significantly enriched category and identifying 339 genes associated with signaling-related protein families (**Figure 3b**; **Supplementary Table 6**). To identify regulatory modules underlying drought response, we performed weighted gene co-expression network analysis (WGCNA) on filtered transcriptome data (mean FPKM > 10). A ‘yellow’ module emerged, exhibiting a robust positive correlation with drought tolerance (r = 0.95, P < 0.01). This module comprised 1027 genes that were largely quiescent under control conditions but strongly induced by drought (**Figure 3a**; **Supplementary Table 7**). Filtering for hub genes with high eigengene-based connectivity (kME > 0.8) and intersecting these with the identified DEGs yielded 395 core drought−responsive genes (**Supplementary Table 8**). To further refine candidate genes, we intersected these 395 core genes with the most significantly enriched GO and KEGG pathways. This analysis identified 38 GO−associated core genes, 38 KEGG−associated core genes, and 4 genes shared between both pathways. These genes formed distinct drought−responsive co-expression networks (**Figure 4**; **Supplementary Table 9**).

### 3.4 Candidate gene prioritization using BSA-seq, RNA-seq, and parental resequencing

To systematically identify candidate drought−resistance genes, we integrated BSA-Seq, RNA-Seq, and parental resequencing data e. A total of 2,850 genes located within the 80 QTL intervals were extracted from the reference genome. Based on transcriptome data, 1621 genes lacking detectable expression were removed. Another 572 genes containing promoter variants but not classified as DEGs were excluded. In addition, genes whose allelic variation patterns were inconsistent with the parental genotype from which the QTL was mapped were also excluded. Finally, subsequent filtering based on functional annotation eliminated genes unrelated to drought-resistance processes, resulting in 41 remaining high−confidence candidate genes, including 21 genes located within major QTLs (**Supplementary Tables 10**, **11**). Comparison with GO−derived co−expression networks identified CM11G00723, CM05G00371, and the transcription factor CM05G00030 as overlapping candidates. Similarly, KEGG−based network comparison identified CM05G02083, CM06G01757, and CM05G00287. Remarkably, CM06G01757, encoding the Ras-related protein gene *CmRABE1c*, was also located within the major QTL M06.42 interval and co-expressed with multiple DEGs, highlighting it as a strong candidate gene for drought tolerance (**Figure 4**).

### 3.5 Functional validation of the candidate gene *CmRABE1c*

**Figure 5:**
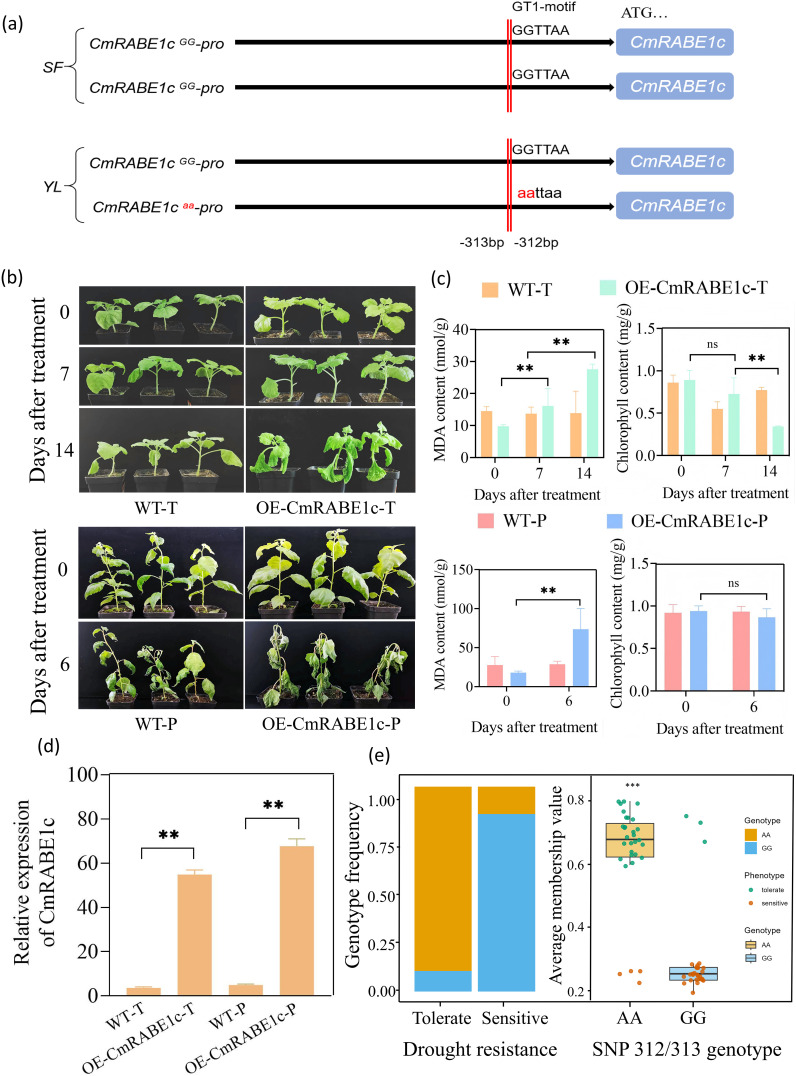
*Cm*RABE1c functions as a negative regulator of drought tolerance, and its promoter SNP is associated with drought responses in Chinese chestnut. **(a)** Schematic representation of the *Cm*RABE1c promoter. The red line marks the SNP locus, where a GG-to-AA substitution disrupts the GT1-motif (GGTTAA) cis-element in ‘YL’. **(b)** Drought phenotypes of *Cm*RABE1c-overexpressing tobacco and poplar plants compared with wild-type controls at the indicated days during drought treatment. **(c)** Physiological responses of WT and OE-*Cm*RABE1c plants under drought stress. **(d)** RT-qPCR quantification of *Cm*RABE1c transcript levels in transgenic tobacco and poplar lines. **(e)** Association analysis between the -312/-313 bp SNP genotypes and drought tolerance in a natural population of 60 chestnut accessions. The symbol ** indicates P < 0.01, *** indicates P < 0.001, and ns means no significant difference (P > 0.05).

Through the above integrated multi−omics analyses, *CmRABE1c* (CM06G01757) was predicted as a key candidate gene associated with drought tolerance. To validate the *CmRABE1c* function on drought resistance, we examined the drought phenotypes and physiological indicators of transgenic tobacco and poplar. The *CmRABE1c*-overexpressing tobacco lines (OE-CmRABE1c-T) exhibited pronounced leaf−edge curling and severe wilting after 7 days of drought, which intensified by day 14 when compared to the empty vector control (WT-T). Physiologically, MDA levels were significantly higher in OE-CmRABE1c-T compared to WT-T at both 7 and 14 days post drought treatment (dpt). Chlorophyll content did not differ between genotypes at 0 and 7 dpt, but was significantly reduced in OE-CmRABE1c-T at 14 dpt. Consistent results were observed in transgenic poplar plants overexpressing CmRABE1c (OE-CmRABE1c-P), which exhibited increased drought sensitivity. At 6 dpt, OE-CmRABE1c-P plants displayed more severe wilting than WT-P controls. MDA content was significantly higher in OE-CmRABE1c-P compared to WT-P at 6 dpt, whereas chlorophyll content did not differ significantly between genotypes at either 0 or 6 dpt (**Figures 5b, c**; **Supplementary Tables 12**, **13**). RT-qPCR confirmed significantly elevated *Cm*RABE1c transcript levels in both transgenic tobacco and poplar relative to their respective wild types (**Figure 5d**). Taken together, our results support that *CmRABE1c* may function as a negative regulator of drought tolerance in Chinese chestnut.

### 3.6 Natural variation in the *CmRABE1c* promoter correlates with drought response

Two consecutive SNPs at positions -312 and -313 bp within the *Cm*RABE1c promoter were detected in the drought-tolerant chestnut accession ‘YL’, but were absent in the drought-sensitive accession ‘SF’. PlantCARE analysis revealed that this GG→AA substitution disrupts the GT1-motif (GGTTAA) cis-element, consistent with the observed drought−induced upregulation of *CmRABE1c* expression (**Figure 5a**, **Supplementary Figure 3**; **Supplementary Table 4**). To further validate the association between this promoter variant and drought tolerance, we genotyped the -312/-313 bp SNP in a panel of 60 natural chestnut accessions exhibiting contrasting drought responses. Genotype frequency analysis revealed that the AA genotype (which disrupts the GT1-motif) was significantly enriched in drought-tolerant accessions, whereas the GG genotype (with an intact GT1-motif) predominated in drought-sensitive accessions. Accessions carrying the AA haplotype consistently displayed substantially higher drought tolerance than those with the GG genotype, indicating that the AA haplotype is strongly associated with enhanced drought tolerance in natural populations. These findings further support the negative regulatory role of CmRABE1c in chestnut drought tolerance (**Figure 5e**; **Supplementary Table 14**).

## 4 Discussion

### 4.1 Drought tolerance in Chinese chestnut is a polygenic quantitative trait

Our two−year drought trials demonstrated that drought injury indices in the F_1_ chestnut seedling population followed a normal distribution, a hallmark of polygenic quantitative traits. This pattern aligns with findings in other perennial woody plants such as apple and poplar, where drought tolerance is governed by multiple loci of varying effect sizes (Wang et al., 2018; Du et al., 2022). Here, two extreme bulks were constructed using 50 individuals each selected based on drought injury index, MDA, H_2_O_2,_ and photosynthetic rate, effectively reducing phenotypic noise. High-quality sequencing with >89.93% mapping rates ensured reliable variant detection and QTL localization. In total, 80 drought-associated QTLs were mapped across all 12 chromosomes, with major-effect QTLs clustered on Chr5 and Chr9. These results reinforce the polygenic nature of drought tolerance in chestnut. BSA-seq has been widely applied to dissect quantitative trait QTLs in woody plants, and our sequencing quality and mapping results were comparable to those reported in other tree species (Liu et al., 2020; Shen et al., 2022). This work reports several genome-wide QTLs associated with drought tolerance in Chinese chestnut, addressing the lack of systematic genetic dissection of drought tolerance in this species and establishing a solid foundation for subsequent candidate gene identification and marker development.

### 4.2 Enrichment of drought-responsive genes in suberin biosynthesis and ABA signaling pathways

Differential expression and enrichment analyses revealed that drought−responsive genes in Chinese chestnut were significantly enriched in pathways related to osmotic adjustment, ABA signal transduction, and response to water deficit. In addition, cell wall-related pathways involved in cellulose and suberin biosynthesis were strongly activated, consistent with the conserved drought−adaptation mechanisms across diverse plant species (de Silva et al., 2021; Keret et al., 2024). In our study, 332 genes were enriched in the suberin biosynthesis pathway, highlighting cell wall modification as a key adaptive strategy enabling chestnut trees to cope with drought in hilly environments. Under drought conditions, the massive accumulation of lignin helps restrict radial water loss between roots and soil to sustain water status, a process known to be induced by ABA signaling (Wang et al., 2014; Zhang et al., 2020; Guo et al., 2023). Notably, hub genes within the drought-tolerance-associated WGCNA yellow module were also enriched in suberin biosynthesis and ABA signaling pathways, forming a core regulatory network between hormonal signals and cell wall modifications underlying chestnut drought response.

### 4.3 Integrated multi-omics analysis enables precise identification of key drought-tolerance genes

Multi-omics integration effectively overcomes the inherent limitations of single-omics strategies when dissecting complex drought tolerance traits, especially in perennial woody plants with large and complex genomes (Zhang et al., 2020; Zeng et al., 2024). In this study, the synergistic integration of BSA-seq, RNA-seq, and parental resequencing circumvented the low resolution and high false-positive rates associated with conventional gene mining methods. BSA-seq identified 80 genome-wide drought-related QTLs, providing a broad genomic framework. RNA-seq subsequently refined candidates by integrating differential expression profiling and WGCNA co-expression network analysis, thereby linking genomic loci to functionally relevant drought-responsive genes. Parental resequencing further validated genetic variants in promoter and coding regions, and excluded genes inconsistent with parental genetic backgrounds. This layered multi-omics workflow reduced the initial 2,850 QTL−associated genes to 41 high−confidence candidates, ultimately identifying *Cm*RABE1c as a critical negative regulator of drought tolerance, confirming the reliability of this strategy. Our work demonstrates that multi-omics integration constitutes a powerful approach for key gene discovery and offers a valuable reference for drought resistance research and molecular breeding in other nut tree species.

### 4.4 *CmRABE1c* is a negative regulator of drought tolerance in Chinese chestnut

Ras-related GTPases are widely implicated bidirectional regulation of stress responses through multiple pathways, such as ROS modulation and stomatal control (Ambastha et al., 2021; Choudhury et al., 2021; Zhang et al., 2023; Puli et al., 2024). Previous studies have shown that *RABE1* (a ras-related protein gene) loss-of-function mutants exhibit reduced ABA sensitivity, impaired stomatal closure, and decreased drought tolerance; conversely, overexpression of *RABE1* significantly enhances drought tolerance in Arabidopsis and Chinese cabbage (Chen et al., 2021). In contrast, the Rab GTPase RABA1E negatively regulates drought resistance in Arabidopsis through the promotion of BPL2 vesicular transport and degradation (Liu et al., 2025). In our study, *CmRABE1c* acts as a critical negative regulator of drought tolerance in Chinese chestnut, as confirmed by multi-omics integration and functional validation. The severe oxidative damage (MDA) and chlorophyll loss observed in *CmRABE1c*-overexpressing lines, together with its transcriptional enrichment in suberin biosynthesis and ABA signaling pathways, suggest that *CmRABE1c* may confers negative regulation of drought tolerance via ABA-mediated cell wall modification and antioxidant pathways.

### 4.5 The *CmRABE1c* promoter SNP312/313 is significantly associated with drought resistance

Diagnostic or functional markers, derived from causal coding or promoter variants, are powerful tools for marker-assisted selection (Xiang et al., 2017; Salgotra and Stewart, 2020). In this study, natural variation analysis revealed that the GG-to-AA substitution in the *Cm*RABE1c promoter disrupts the GT1-motif and is strongly associated with enhanced drought tolerance. Given that the AA genotype is dominant among tolerant accessions and the polymorphism involves a clear dinucleotide substitution (GG-to-AA), this locus is ideally suited for KASP assay design. Compared to traditional gel-based methods such as CAPS or direct sequencing, the KASP platform offers high-throughput, cost-effectiveness, and excellent accuracy, making it suitable for large-scale genotyping in commercial breeding pipelines (Semagn et al., 2014). This SNP represents a robust functional marker, laying a foundation for precise molecular breeding of drought-resistant chestnut varieties.

### 4.6 Future perspectives and limitations

Despite the valuable insights provided by our study, several limitations should be acknowledged. First, QTL mapping was confined to a single F_1_ segregating population derived from the cross between ‘Yanlong’ and ‘Shuofeng’; consequently, the identified loci reflect only the allelic diversity present between these specific parents. Multi-population validation is therefore required to confirm the stability and universality of these QTLs across diverse genetic backgrounds. Second, functional validation in stable transgenic chestnut lines and the mechanistic interpretation of how *CmRABE1c* affects drought signaling pathways remains to be elucidated. Third, the diagnostic SNP marker was evaluated using a panel of 60 accessions; broader genotyping across expanded and more diverse germplasm collections is warranted to fully establish its utility for marker-assisted selection. Addressing these limitations will be the focus of our subsequent research to facilitate the genetic improvement of drought tolerance in Chinese chestnut.

## Conclusion

This study integrated BSA-seq and RNA-seq to identify 80 drought-tolerance-associated QTLs in Chinese chestnut. Combined multi-omics analysis and heterologous transgenic verification revealed that CmRABE1c likely acts as a key negative regulator governing drought responses in chestnut. A promoter SNP (312/313) in *CmRABE1c* was significantly associated with drought resistance. These findings provide valuable functional markers and genetic resources for marker-assisted selection, supporting the advancement and applications of genomic breeding for drought tolerance in Chinese chestnut.

## Data Availability

The raw sequence data reported in this paper have been deposited in the Genome Sequence Archive (Genomics, Proteomics & Bioinformatics 2025) in National Genomics Data Center (Nucleic Acids Res 2025), China National Center for Bioinformation / Beijing Institute of Genomics, Chinese Academy of Sciences (GSA: CRA046670) that are publicly accessible at https://ngdc.cncb.ac.cn/gsa.
